# The DLG1-AS1/miR-497/YAP1 axis regulates papillary thyroid cancer progression

**DOI:** 10.18632/aging.104121

**Published:** 2020-11-16

**Authors:** Yong Huang, KeWei Zhang, Yinghua Li, Yuyin Dai, Hongguang Zhao

**Affiliations:** 1 Department of Nuclear Medicine, The First Hospital of Jilin University, Changchun 130021, China; 2 Department of Thoracic Surgery, The First Hospital of Jilin University, Changchun 130021, China

**Keywords:** papillary thyroid cancer, DLG1-AS1, miR-497, YAP1, lncRNA

## Abstract

The long non-coding RNA (lncRNA), DLG1-AS1, is upregulated in papillary thyroid cancer (PTC) tissues and cell lines. Here, we found that increased expression of DLG1-AS1 caused lymph node metastasis and advanced tumor-node-metastasis (TNM) stage. DLG1-AS1 knockdown inhibited proliferation, invasion, and migration of PTC cells, and impaired tumorigenesis *in vivo* in mouse xenografts. DLG1-AS1 functions as a competing endogenous RNA (ceRNA) for miR-497. Further investigation revealed that DLG1-AS1 regulated yes-associated protein 1 (YAP1; a known target of miR-497) by competitively binding to miR-497. Moreover, inhibition of miR-497 abrogated the inhibitory effects of DLG1-AS1 depletion on PTC cells. These findings demonstrate that the DLG1-AS1–miR-497–YAP1 axis promotes the growth and metastasis of PTC by forming a ceRNA network.

## INTRODUCTION

Papillary thyroid cancer (PTC) is one of the common endocrine malignancies that accounts for approximately 80% of thyroid cancers worldwide [1]. Recent years have witnessed a continuous increase in the incidence of PTC. Moreover, despite the favorable prognosis of patients with early-stage PTC, the 5-year survival rate of those with advanced PTC is poor due to cancer metastasis and proliferation [2, 3].

Abnormal expression of long non-coding RNAs (lncRNAs) is known to be involved in the occurrence and development of several cancers [4, 5]. LncRNAs—a type of RNAs with a length of more than 200 nucleotides and limited protein-coding ability—are involved in several cellular processes, such as inflammatory and stress responses, proliferation, differentiation, apoptosis, and invasion [6, 7]. For example, numerous lncRNAs are implicated in proliferation, invasion, and apoptosis of PTC. Thus, these could serve as a diagnostic marker and therapeutic target for PTC [8–10].

DLG1-AS1 (ENSG00000227375), a recently discovered lncRNA, has an oncogenic function in cervical cancer [11]. A recent study demonstrated that plasma lncRNA DLG1-AS1 is upregulated in patients with PTC as compared to those with benign thyroid nodules and healthy controls. In addition, the overexpression of DLG1-AS1 promotes PTC proliferation by downregulating miR-199a-3p [12]. Here, we investigated the function and mechanism of DLG1-AS1 in PTC migration and invasion using a series of *in vivo* and *in vitro* experiments.

## RESULTS

### Overexpressed DLG1-AS1 is associated with poor clinical outcomes in PTC patients

**Figure 1:**
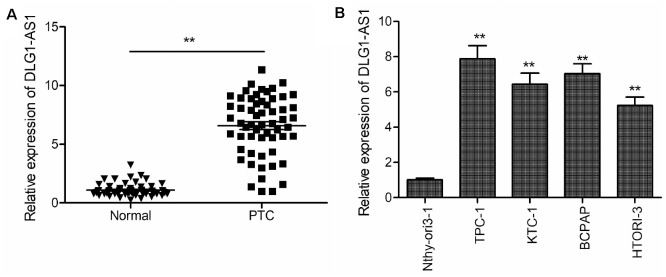
**DLG1-AS1 expression is upregulated in PTC tissues and cell lines.** (**A**) Quantitative real-time reverse transcription-polymerase chain reaction (qRT-PCR) assays showing upregulated expression of DLG1-AS1 in PTC tissues as compared with that in adjacent normal tissues. (**B**) The qRT-PCR assays reveal upregulated expression of DLG1-AS1 in four PTC cell lines as compared with that in a normal thyroid epithelial cell line (Nthy-ori 3-1). All experiments were performed in triplicate, and data are expressed as mean ± standard deviation (SD) (*P < 0.05, **P < 0.01).

The expression of DLG1-AS1 was determined in PTC tissues and adjacent non-tumor tissues (*n* = 58) using quantitative real-time polymerase chain reaction (qRT-PCR). The results revealed that compared with adjacent non-tumor tissues, DLG1-AS1 was upregulated in PTC tissues (Figure 1A). We further found that expression of DLG1-AS1 in tumor tissues positively correlated with patients’ tumor-node metastasis (TNM) stage (*P* = 0.0155) and lymph node metastasis (*P* = 0.0125). However, it had no association with gender (*P* = 0.288), age (*P* = 0.592), and tumor size (*P* = 0.1744) (Table 1). In addition, we found that compared with a normal thyroid epithelial cell line (Nthy-ori 3-1), the expression of DLG1-AS1 was markedly increased in the four PTC cell lines (Figure 1B). These results implied that DLG1-AS1 might participate in the progression of PTC.

**Table 1:** Correlation between clinicopathological features and DLG1-AS1 expression in PTC tissues.

**Variables**	**No. of cases**	**DLG1-AS1 expression**		***P* value**
		**High**	**Low**	
Age (years)				*P* = 0.2880
<60	24	11	13	
≥60	34	21	13	
Gender				*P* = 0.5920
Male	23	14	9	
Female	35	18	17	
TNM stage				*P* = 0.0155
T1-T2	47	22	25	
T3-T4	11	10	1	
Tumor size				*P* = 0.1744
<1	36	17	19	
≥1	22	15	7	
Lymph node metastasis				*P* = 0.0125
No	44	20	24	
Yes	14	12	2	

Note: Fifty-eight patients were split into two groups based on the median value of DLG1-AS1 in PTC tissues: DLG1-AS1 high group (*n* = 32) and DLG1-AS1 low group (*n* = 26). PTC, papillary thyroid cancer; TNM, tumor-node metastasis.

### Knockdown of DLG1-AS1 inhibited cell viability and invasion of PTC cells

**Figure 2:**
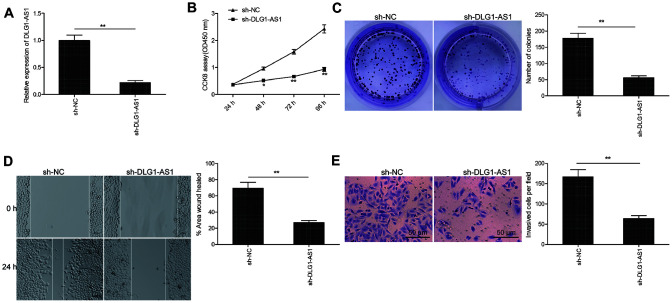
**Knockdown of DLG1-AS1 inhibited cell viability and invasion of PTC cells.** (**A**) The expression of DLG1-AS1 is downregulated in TPC-1 cells transfected with sh-DLG1-AS1 or sh-NC. (**B**–**E**) Knockdown of DLG1-AS1 inhibits proliferation, colony formation, migration, and invasion in TPC-1 cells transfected with sh-DLG1-AS1 or sh-NC. All experiments were performed in triplicate, and data are expressed as mean ± standard deviation (SD) (*P < 0.05, **P < 0.01).

To identify the function of DLG1-AS1 in tumorigenesis, we determined multiple parameters including proliferation, migration, and invasion in TPC-1 (human PTC cell line) cells. Initially, we knocked down DLG1-AS1 in TPC-1 cells by transfecting the cells with shRNA against DLG1-AS1 (sh-DLG1-AS1). The qRT-PCR results confirmed a highly downregulated expression of DLG1-AS1 post silencing in TPC-1 cells (Figure 2A). Next, the CCK-8 and colony formation assays were performed to observe the effect of DLG1-AS1 depletion on the proliferation of PTC cells. The CCK-8 assay demonstrated reduced cell viability of TPC-1 cells at 48, 72, and 96 h following DLG1-AS1 depletion (Figure 2B). Consistent with this result, knockdown of DLG1-AS1 inhibited colony formation ability of TPC-1 cells (Figure 2C). Next, we performed studies to check the migration and invasion capacities of TPC-1 cells transfected with sh-DLG1-AS1. We observed that the knockdown of DLG1-AS1 decreased the migration and invasion abilities of TPC-1 cells (Figure 2D, 2E). These data confirmed that knockdown of DLG1-AS1 inhibited cell proliferation, migration, and invasion of PTC cells.

### DLG1-AS1 functions as a molecular sponge for miR-497 in PTC cells

**Figure 3:**
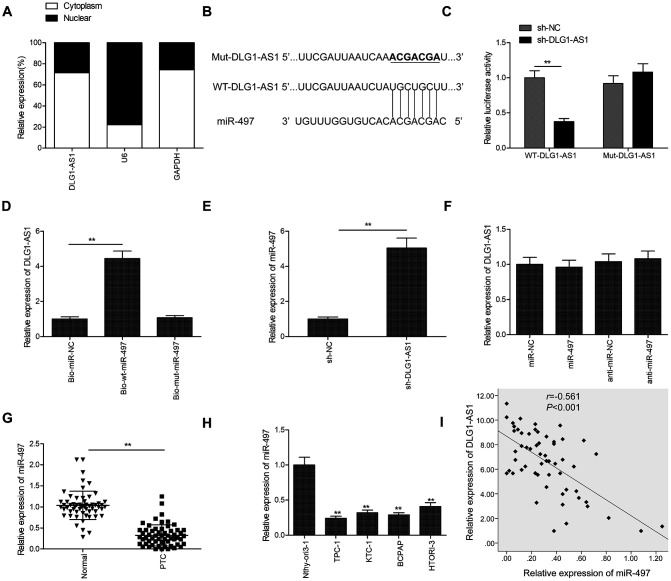
**DLG1-AS1 functions as a molecular sponge for miR-497 in PTC cells.** (**A**) The expression of DLG1-AS1 was determined in cytoplasmic and nuclear fractions of TPC-1 and B-CPAP cells. (**B**) The predicted binding site and mutant sites between DLG1-AS1 and miR-497 are shown. (**C**) Dual-luciferase reporter assay revealed that the overexpression of miR-497 negatively regulated the luciferase activity of DLG1-AS1-WT, rather than DLG1-AS1-Mut. WT: wild-type, Mut: mutant type. (**D**) The association between DLG1-AS1 and miR-497 was determined in TPC-1 cells by RNA pull-down assay. (**E**) Increased expression of miR-497 in TPC-1 cells transfected with sh-DLG1-AS1 or sh-NC. (**F**) The expression of DLG1-AS1 was determined in TPC-1 cells transfected with miR-497 mimics or miR-NC. (**G**) The expression of miR-497 is downregulated in PTC tissues as compared with that in adjacent normal tissues. (**H**) Quantitative real-time reverse transcription-polymerase chain reaction (qPCR) assays showing reduced expression of miR-497 in four PTC cell lines as compared with that in a normal thyroid epithelial cell line (Nthy-ori 3-1). (**I**) Analysis of correlation between DLG1-AS1 and miR-497 expression in PTC tissues by Pearson’s correlation. All experiments were performed in triplicate, and data are expressed as mean ± standard deviation (SD) (*P < 0.05, **P < 0.01).

The subcellular localization of an lncRNA represents its biological function. To determine the cellular localization of DLG1-AS1, we isolated the cytoplasmic and nuclear fractions of TPC-1 cells, with glyceraldehyde 3-phosphate dehydrogenase (GAPDH) as the cytoplasmic control and U6 as the nuclear control, respectively. The results of qRT-PCR demonstrated that 71.2% of the DLG1-AS1 was present in the cytoplasmic fraction of TPC-1 cells (Figure 3A). Given that DLG1-AS1 is primarily located in the cytoplasmic fraction of PTC cells, we hypothesized that DLG1-AS1 could serve as a competing endogenous RNA (ceRNA) for sponging miRNAs in PTC. The bioinformatics prediction database (Starbase2.0; http://starbase.sysu.edu.cn/) showed a shared binding site for miR-497 in the DLG1-AS1 molecule (Figure 3B). Based on these binding sequences, we constructed DLG1-AS1-WT and DLG1-AS1-MUT plasmids and performed luciferase activity assays. The results demonstrated that the overexpression of miR-497 decreased the luciferase activity of DLG1-AS1-WT. However, it had no effect on DLG1-AS1-MUT in TPC-1 and B-CPAP cells (Figure 3C). The RNA-pull down assay further confirmed the binding of DLG1-AS1 to miR-497 in TPC-1 cells (Figure 3D). In addition, we found that the knockdown of DLG1-AS1 increased the expression of miR-497 (Figure 3E), whereas the upregulation or downregulation of miR-497 in PTC cells did not alter the expression of DLG1-AS1 (Figure 3F). Furthermore, we found reduced expression of miR-497 in PTC tissues (Figure 3G) and cell lines (Figure 3H). Besides, the expression of DLG1-AS1 reversely correlated with the expression of miR-497 in PTC tissues (Figure 3I). These results showed that miR-497 could bind to DLG1-AS1 *in vitro*.

### MiR-497 inhibition reverses the inhibitory effects of DLG1-AS1 knockdown in PTC cells

**Figure 4:**
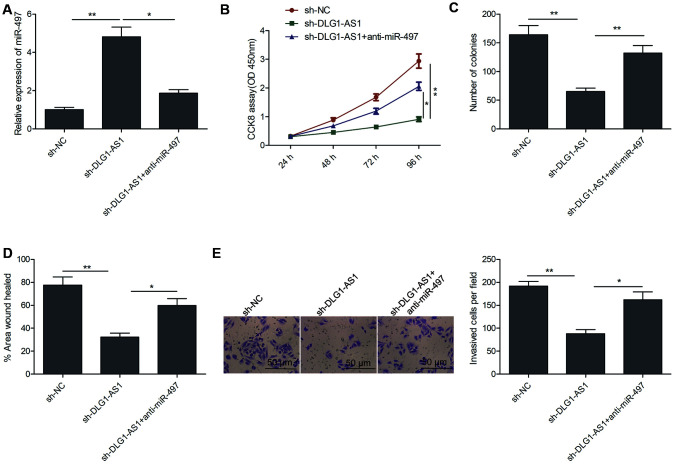
**MiR-497 inhibition reversed the inhibitory effects of DLG1-AS1 knockdown in PTC cells.** (**A**)The expression of miR-497 was assessed in TPC-1 cells transfected with sh-NC, sh-DLG1-AS1, and sh-DLG1-AS1+miR-497 inhibitor (anti-miR-497). (**B**–**E**) Inhibition of miR-497 partially reversed the inhibitory effects of DLG1-AS1 knockdown on cell proliferation, colony formation, migration, and invasion in TPC-1 cells. All experiments were performed in triplicate, and data are expressed as the mean ± standard deviation (SD) (*P < 0.05, **P < 0.01).

Next, we performed rescue experiments to study the involvement of miR-497 in the effects of DLG1-AS1 on cell proliferation, colony formation, migration, and invasion of PTC cells. The results of qRT-PCR revealed that miR-497 inhibitor decreased DLG1-AS1 depletion-induced expression of miR-497 in TPC-1 cells (Figure 4A). Rescue experiments revealed that the downregulation of miR-497 partially reversed the effect of DLG1-AS1 depletion on cell proliferation, colony formation, migration, and invasion in TPC-1 cells (Figure 4B–4E). These results implied that DLG1-AS1 exerted a tumor-promoting effect on PTC cells by functioning as a ceRNA for sponging miR-497.

### DLG1-AS1 regulates *YAP1*, the target gene of miR-497 in PTC cells

**Figure 5:**
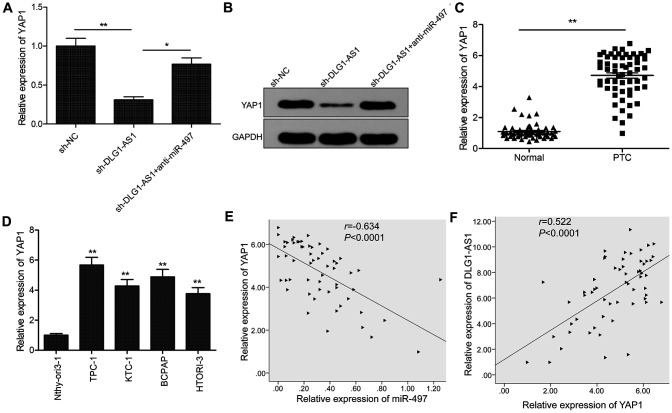
**DLG1-AS1 regulates *YAP1*, the target gene of miR-497 in PTC cells.** (**A**, **B**) The expression of YAP1 was examined in TPC-1 cells transfected with sh-NC, sh-DLG1-AS1, and sh-DLG1-AS1+miR-497 inhibitor (anti-miR-497). (**C**) Compared with adjacent normal tissues, the expression of YAP1 increased in PTC tissues. (**D**) Quantitative real-time polymerase chain reaction (qPCR) assays showing upregulated expression of YAP1 in four PTC cell lines as compared with that in a normal thyroid epithelial cell line (Nthy-ori 3-1). (**E**) Analysis of correlation between YAP1 and miR-497 expression in PTC tissues by Pearson’s correlation analysis. (**F**) Analysis of correlation between YAP1 expression and DLG1-AS1 expression in PTC tissues by Pearson’s correlation analysis. All experiments were performed in triplicate, data are expressed as mean ± standard deviation (SD) (*P < 0.05, **P < 0.01).

A previous study showed that miR-497 could target YAP1 to inhibit PTC progression [13]. Here, we investigated the association of DLG1-AS1, miR-497, and YAP1. We found that knockdown of DLG1-AS1 inhibited the expression of *YAP1* in TPC-1 cells, whereas miR-497 inhibitor could partially rescue this effect (Figure 5A, 5B). Moreover, we found that the expression of *YAP1* was upregulated in PTC tissues and cell lines (Figure 5C, 5D). Pearson’s correlation analysis indicated that the expression of *YAP1* was negatively correlated with that of miR-497 (Figure 5E) and positively correlated with that of DLG1-AS1 in PTC tissues (Figure 5F). These data indicated that DLG1-AS1 regulated the expression of *YAP1* by sponging miR-497 in PTC.

### DLG1-AS1 knockdown suppresses tumor growth in PTC xenograft model

**Figure 6:**
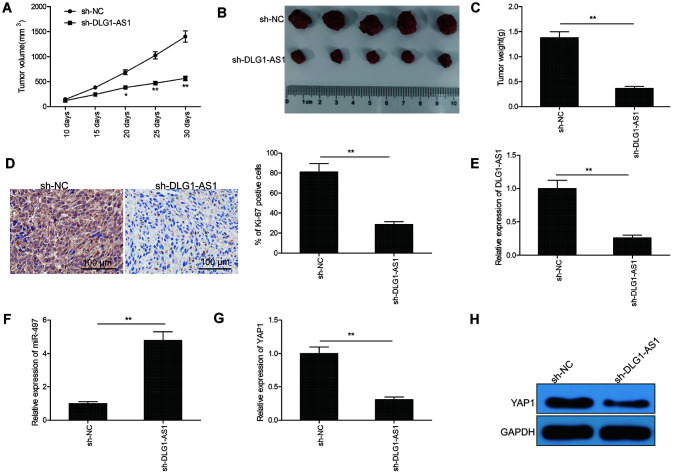
**Knockdown of DLG1-AS1 suppressed tumor growth in PTC xenograft model.** (**A**) Tumor volumes were examined every 7 days until the mice were sacrificed. (**B**) Tumor image was captured at the end of experiments. (**C**) Tumor weight was measured at the end of experiments. (**D**) The expression of Ki-67 was determined in xenograft tumor by immunohistochemistry (IHC). (**E**) The expression of DLG1-AS1 was determined in xenograft tumor by quantitative real-time polymerase chain reaction (qRT-PCR). (**F**)The expression of miR-497 was examined in xenograft tumor by qRT-PCR. (**G**, **H**) The mRNA and protein expression of YAP1 measured in xenograft tumors. All experiments were performed in triplicate and data are expressed as the mean ± standard deviation (SD) (*P < 0.05, **P < 0.01).

To explore whether the expression of DLG1-AS1 affected tumorigenesis, TPC-1 cells infected with lenti-sh-DLG1-AS1 or lenti-sh-NC were used in a BALB/c nude mouse xenograft model. The data showed delayed tumor growth on days 20, 25, and 30 post lenti-sh-DLG1-AS1 injection in comparison with lenti-sh-NC treatment (Figure 6A). Thirty days after the injection, all mice were sacrificed, and the tumor tissues were stripped and weighed. The weight and size of the tumor were smaller in the lenti-sh-DLG1-AS1 group than in the lenti-sh-NC group (Figure 6B, 6C). Furthermore, immunohistochemistry (IHC) assay showed that mice treated with lenti-sh-DLG1-AS1 had lower expression of Ki-67, a proliferation-specific marker (Figure 6D). To confirm the expression of DLG1-AS1, miR-497, and *YAP1* in tumor xenograft, qRT-PCR was performed on tissues at the end of the experiments. Results showed that the depletion of DLG1-AS1 decreased the expression of DLG1-AS1 (Figure 6E), increased the expression of miR-497 (Figure 6F), and reduced that of YAP1 mRNA and protein (Figure 6G, 6H) in xenograft tissues, suggesting that the knockdown of DLG1-AS1 efficiently suppressed tumor growth *in vivo* by regulating the miR-497/YAP1 axis.

## DISCUSSION

Several studies have suggested that lncRNAs function as oncogenic or tumor-suppressive molecules in PTC progression and contribute to the tumorigenesis and metastasis of PTC [8–10]. For example, lncRNA SNHG22 functions as an oncogene and promotes the malignancy of PTC by increasing the expression of *ZEB1* by competitively binding to miRNA-429 [14]. Similarly, lncRNA ASMTL-AS1 serves as a tumor suppressor that inhibits PTC growth and glycolysis by regulating the miR-93-3p/miR-660/FOXO1 axis [15]. LncRNA LINC00520 contributes to PTC progression by serving as a ceRNA of miRNA-577 to increase the expression of *Sphk2* [16]. In the present study, the qRT-PCR results confirmed that DLG1-AS1 was markedly upregulated in PTC tissues—a finding consistent with that reported in a previous study [12]. Besides, increased expression of DLG1-AS1 was linked to the TNM stage and lymph node metastasis in patients with PTC.

DLG1-AS1, a novel lncRNA, was reported to promote the proliferation of cervical cancer cells by competitively binding to miR-107 and upregulating the expression of *ZHX1* [11]. Recently, a study showed that DLG1-AS1 was upregulated in the plasma of patients with PTC [12]. We showed that the knockdown of DLG1-AS1 inhibited PTC cell viability, migration, and invasion. These phenotypes were further verified using an *in vivo* xenograft mice model, in which the knockout of DLG1-AS1 suppressed PTC tumorigenesis. These results suggested the oncogenic function of DLG1-AS1 in PTC.

Cytoplasmic lncRNAs function as ceRNAs and regulate mRNA expression by competitively binding with miRNAs [17, 18]. Cytoplasmic and nuclear fractionation assay studies have shown that DLG1-AS1 is primarily expressed in the cytoplasm of TPC-1 cells. Therefore, we postulated that DLG1-AS1 might perform its tumorigenic function by functioning as a ceRNA for sponging miRNAs. To understand how depletion of DLG1-AS1 inhibited PTC cell proliferation and invasion, the Starbase2.0 database was used to select potential miRNAs that could bind with miRNAs. Luciferase and RNA pull-down assays confirmed the binding of miR-497 to DLG1-AS1. Previous studies have demonstrated that miR-497 functions as a tumor suppressor in PTC [13, 19–21]. We discovered that miR-497 inhibition partially reversed the inhibitory effect of DLG1-AS1 depletion in TPC-1 cells, suggesting that DLG1-AS1 functions as a ceRNA for miR-497 in PTC.

YAP1, a transcriptional co-activator that functions in the Hippo signaling pathway [22, 23], is involved in the initiation and development of several cancers including PTC [24–26]. Previous studies have revealed that YAP1 is a direct target of miR-497 in PTC [13]. Thus, we investigated the association between DLG1-AS1, miR-497, and YAP1 in PTC. We found that the knockdown of DLG1-AS1 reduced the expression of YAP1, whereas inhibition of miR-497 partially reversed this effect. Further experiments confirmed that the expression of YAP1 negatively correlated with that of miR-497, and positively correlated with that of DLG1-AS1 in PTC tissues. These results suggested that DLG1-AS1 regulated the expression of YAP1 by sponging miR-497.

In summary, the upregulation of DLG1-AS1 correlated with worse clinical outcomes and prognosis in patients with PTC. Knockdown of DLG1-AS1 inhibited cell proliferation, invasion, and migration *in vitro*, and retarded tumor growth *in vivo* by regulating the miR-497/YAP1 axis. These results suggest that DLG1-AS1 could be used as a useful therapeutic target for PTC.

## MATERIALS AND METHODS

### Patients and thyroid tissue samples

Biopsy samples of PTC tissues and paracancerous tissues were obtained from 58 patients who underwent surgical resection between January 2018 and January 2019. After surgery, all samples were immediately frozen and stored in liquid nitrogen until use. All patient-derived information was recorded following the protocols approved by the ethical standards of the Ethics Committee of the First Hospital of Jilin University (Changchun, China). Written informed consent was obtained from all participants.

### Cell culture and transfection

Four PTC cell lines (TPC1, KTC-1, B-CPAP, and HTori-3) and a normal thyroid epithelial cell line (Nthy-ori 3-1) were bought from Cell Bank of Chinese Academy of Science (Shanghai, China). The cells were grown in RPMI-1640 (GIBCO-BRL, CA, USA) supplemented with 10% fetal bovine serum (FBS; GIBCO-BRL), 100 mg/mL streptomycin and 100 U/mL penicillin (Invitrogen; Thermo Fisher Scientific, Inc.). For the downregulation of DLG1-AS1, recombinant lentivirus short-hairpin RNA plasmid directly targeting DLG1-AS1 (sh-DLG1-AS1) and corresponding negative control scramble (sh-NC) plasmids were synthesized by GenePharma (Shanghai, China). TPC-1 cells were transfected with sh-DLG1-AS1 and sh-NC lentiviral transduction particles (MOI = 20) using 5 μg/mL polybrene (GeneChem). Stably transfected cells were obtained using 1 μg/mL puromycin (Calbiochem, USA).

MiR-497 inhibitor, miR-497 mimics, and their controls were obtained from GenePharma (Shanghai, China). TPC-1 cells were transfected with these molecules using Lipofectamine 3000 (Invitrogen; Carlsbad, CA) following the manufacturer’s protocol.

### Quantitative real-time reverse transcription-polymerase chain reaction

Total RNA from tissues and cells was isolated using the miRNeasy Mini Kit (Qiagen, Valencia, CA, USA). Its concentration was assessed by NanoDrop 2000 (Thermo Fisher; Wilmington, DE, USA). Total RNA samples were reverse transcribed using TransScript First-Strand cDNA Synthesis SuperMix (TransGen; Beijing, China). Real-time PCR reactions were performed using SYBR Green qPCR SuperMix (Applied Biosystems Life Technologies; Foster, CA, USA) under ABI Prism 7900 sequence detection system (Applied Biosystems Life Technologies). The primers used in this study have been described previously [11, 13]. The expression was quantified using the 2^−ΔΔCt^ method, and U6 and GADPH were used as internal controls.

### Cell proliferation and colony formation assays

Cell proliferation was determined using the Cell Counting Kit-8 (CCK-8) assay and colony formation assay. For the CCK-8 assay, transfected cells were plated in 96-well plates at a density of 5,000 cells/well and incubated for 24, 48, 72, or 96 h. Next, 10 μL of CCK-8 agent was added to each well, followed by incubation for 2 h and measurement of optical density (OD) value at 450 nm using an enzyme immunoassay analyzer (Bio-Rad; Hercules, CA, USA). All experiments were performed in triplicate.

To perform the colony formation assay, 500 transfected cells were plated in 6-well plates and maintained in RPMI 1640 with 10% FBS for 10 days. The clones were fixed with 4% paraformaldehyde for 30 min and stained with 0.5% crystal violet solution for 30 min at 37° C. The clones were photographed and manually counted.

### Cell migration and invasion assays

Wound healing assay was performed to determine cell migration ability as described previously [27]. Images were captured using a light microscope at 0 h and 24 h after creating the wound. The wound area was determined using the Image J software 3.0.

For invasion assay, 1.0 × 10^5^ transfected cells in 200 μL of serum-free medium were plated into the upper chambers coated with Matrigel. Next, 600 μL of RPMI-1640 medium containing 20% FBS was added to the bottom chamber as a chemoattractant. After a 24-h incubation, invasive cells were fixed with 4% paraformaldehyde for 30 min and stained with 0.5% crystal violet solution for 30 min at 37° C. Five random fields of view were selected to count the cell numbers under a light microscope at a magnification of ×20.

### Subcellular fractionation and localization

Nuclear and cytoplasmic RNAs of TPC-1 cells were extracted using the PARIS kit (Invitrogen) according to the manufacturer’s protocol. The cellular localization of DLG1-AS1 was measured by qRT-PCR analysis of cytoplasmic and nuclear RNAs. U6 was used as an endogenous control for the nucleus, and GAPDH was used as an endogenous control for the cytoplasm.

### Dual-luciferase reporter assay

The interaction between DLG1-AS1 and miR-497 was predicted by Starbase3 v3.0 (http://starbase.sysu.edu.cn/). The DLG1 cDNA fragments containing the predicted miR-497-binding site and the matching mutant were synthesized and inserted into a psiCHECK2 vector (Promega; Madison, WI, USA). These were named as WT-DLG1-AS1 and Mut-DLG1-AS1, respectively. MiR-497 mimics or negative controls (100 nM) and reporter plasmid were transfected into TPC-1 cells using Lipofectamine 2000. Luciferase activity was measured at 48 h using the dual-luciferase reporter assay kit (Promega) following the manufacturer’s protocol.

### RNA pull-down assay

The wt-miR-497 mimics, mut-miR-497 mimics, and miR-NC mimics were biotinylated using the Biotin RNA Labeling Mix (Roche Diagnostics; Indianapolis, IN, USA) according to the manufacturer’s instructions. The biotinylated mimics were transfected into TPC-1 cells and incubated for 48 h. The whole-cell lysates were precipitated with streptavidin-coated magnetic beads and cultured for 3 h at 4° C. The beads were harvested by centrifugation and washed with a wash buffer. The RNA complexes bound to the beads were isolated and purified using an RNeasy Mini kit (Qiagen). The total RNA was determined using qRT-PCR to measure the expression of DLG1-AS1.

### Western blotting

Total protein was isolated using the radioimmunoprecipitation assay (RIPA) protein extraction reagent (Beyotime; Beijing, China) containing a protease inhibitor and phenylmethylsulfonyl fluoride. After determining the protein concentration, approximately 30 μg of the protein extract was separated by 10% sodium dodecyl sulfate-polyacrylamide gel electrophoresis (SDS-PAGE) and transferred to a nitrocellulose membrane (Sigma-Aldrich). After blocking with 5% skimmed milk, the membrane was incubated with primary antibodies against GAPDH and YAP1 overnight at 4° C. Next, the membrane was incubated with corresponding secondary antibodies (Abcam) for 2 h. All antibodies were purchased from Abcam (USA). The protein bands were observed using an enhanced chemiluminescence detection system (Bio-Rad). The intensities of the bands were observed and determined by Quantity One software (Bio-Rad) with GAPDH as the control.

### Tumor xenografts

Stable TPC-1 cells infected with lentivirus sh-DLG1-AS1 or sh-NC were mixed with Matrigel and intraperitoneally (i.p.) injected into 6-week-old female BALB/c nude mice (each group consisted of five mice; the Laboratory Animal Center of Jilin University). The tumor volume was determined by measuring the tumor width (W) and length (L) using a Vernier caliper every 5 days and calculated using the formula: Volume (mm^3^) = 0.5 × width^2^ × length. Thirty days after the injection, all mice were sacrificed by intraperitoneal injection of 200 mg/kg pentobarbital sodium (Sigma-Aldrich; Merck KGaA). Tumor tissues were stripped, weighed, imaged, and stored at −80° C until use. Animal experiments were conducted in accordance with the regulations for the Administration of Affairs Concerning Experimental Animals (China, 1988), and approved by the Animal Care and Use Committee of the First Hospital of Jilin University.

### Immunohistochemistry

Immunohistochemistry was performed to detect the expression of Ki-67 in xenografted mice tumor tissues as described previously [28].

### Statistical analysis

Experimental data are presented as means ± standard deviations (SDs). Statistical analysis was performed using the SPSS 19.0 software package (SPSS, Inc., Chicago, IL, USA) with Student’s *t*-test or one-way analysis of variance, followed by Bonferroni *post ho*c test or the chi-squared test. Expression correlation assays were analyzed using Spearman’s rank correlation analysis. A value of *P* less than 0.05 was considered significant.

## References

[1] Siegel R, Naishadham D, Jemal A. Cancer statistics, 2013. CA Cancer J Clin. 2013; 63:11–30. 10.3322/caac.21166 23335087

[2] Pemayun TG. Current diagnosis and management of thyroid nodules. Acta Med Indones. 2016; 48:247–57. 27840362

[3] Leboulleux S, Rubino C, Baudin E, Caillou B, Hartl DM, Bidart JM, Travagli JP, Schlumberger M. Prognostic factors for persistent or recurrent disease of papillary thyroid carcinoma with neck lymph node metastases and/or tumor extension beyond the thyroid capsule at initial diagnosis. J Clin Endocrinol Metab. 2005; 90:5723–9. 10.1210/jc.2005-0285 16030160

[4] Corrà F, Agnoletto C, Minotti L, Baldassari F, Volinia S. The network of non-coding RNAs in cancer drug resistance. Front Oncol. 2018; 8:327. 10.3389/fonc.2018.00327 30211115 PMC6123370

[5] Balas MM, Johnson AM. Exploring the mechanisms behind long noncoding RNAs and cancer. Noncoding RNA Res. 2018; 3:108–17. 10.1016/j.ncrna.2018.03.001 30175284 PMC6114262

[6] Iyer MK, Niknafs YS, Malik R, Singhal U, Sahu A, Hosono Y, Barrette TR, Prensner JR, Evans JR, Zhao S, Poliakov A, Cao X, Dhanasekaran SM, et al. The landscape of long noncoding RNAs in the human transcriptome. Nat Genet. 2015; 47:199–208. 10.1038/ng.3192 25599403 PMC4417758

[7] Ponting CP, Oliver PL, Reik W. Evolution and functions of long noncoding RNAs. Cell. 2009; 136:629–41. 10.1016/j.cell.2009.02.006 19239885

[8] Murugan AK, Munirajan AK, Alzahrani AS. Long noncoding RNAs: emerging players in thyroid cancer pathogenesis. Endocr Relat Cancer. 2018; 25:R59–82. 10.1530/ERC-17-0188 29146581

[9] Sedaghati M, Kebebew E. Long noncoding RNAs in thyroid cancer. Curr Opin Endocrinol Diabetes Obes. 2019; 26:275–81. 10.1097/MED.0000000000000497 31385810

[10] Mahmoudian-Sani MR, Jalali A, Jamshidi M, Moridi H, Alghasi A, Shojaeian A, Mobini GR. Long non-coding RNAs in thyroid cancer: implications for pathogenesis, diagnosis, and therapy. Oncol Res Treat. 2019; 42:136–42. 10.1159/000495151 30799425

[11] Rui X, Xu Y, Huang Y, Ji L, Jiang X. lncRNA DLG1-AS1 promotes cell proliferation by competitively binding with miR-107 and up-regulating ZHX1 expression in cervical cancer. Cell Physiol Biochem. 2018; 49:1792–803. 10.1159/000493625 30231238

[12] He T, Wang H, Sun J, Wu J, Gong F, Li S, Wang H, Li Y. Altered expression of DLG1-AS1 distinguished papillary thyroid carcinoma from benign thyroid nodules. BMC Endocr Disord. 2019; 19:122. 10.1186/s12902-019-0440-x 31718630 PMC6852766

[13] Cheng H, Dong H, Feng J, Tian H, Zhang H, Xu L. miR-497 inhibited proliferation, migration and invasion of thyroid papillary carcinoma cells by negatively regulating YAP1 expression. Onco Targets Ther. 2018; 11:4711–21. 10.2147/OTT.S164052 30127619 PMC6091470

[14] Gao H, Sun X, Wang H, Zheng Y. Long noncoding RNA SNHG22 increases ZEB1 expression via competitive binding with microRNA-429 to promote the Malignant development of papillary thyroid cancer. Cell Cycle. 2020; 19:1186–99. 10.1080/15384101.2020.1749466 32306838 PMC7217354

[15] Feng Z, Chen R, Huang N, Luo C. Long non-coding RNA ASMTL-AS1 inhibits tumor growth and glycolysis by regulating the miR-93-3p/miR-660/FOXO1 axis in papillary thyroid carcinoma. Life Sci. 2020; 244:117298. 10.1016/j.lfs.2020.117298 31953163

[16] Guo Q, Xu L, Peng R, Ma Y, Wang Y, Chong F, Song M, Dai L, Song C. Characterization of lncRNA LINC00520 and functional polymorphisms associated with breast cancer susceptibility in Chinese Han population. Cancer Med. 2020; 9:2252–68. 10.1002/cam4.2893 31997582 PMC7064040

[17] Salmena L, Poliseno L, Tay Y, Kats L, Pandolfi PP. A ceRNA hypothesis: the rosetta stone of a hidden RNA language? Cell. 2011; 146:353–58. 10.1016/j.cell.2011.07.014 21802130 PMC3235919

[18] Dey BK, Mueller AC, Dutta A. Long non-coding RNAs as emerging regulators of differentiation, development, and disease. Transcription. 2014; 5:e944014. 10.4161/21541272.2014.944014 25483404 PMC4581346

[19] Wang P, Meng X, Huang Y, Lv Z, Liu J, Wang G, Meng W, Xue S, Zhang Q, Zhang P, Chen G. MicroRNA-497 inhibits thyroid cancer tumor growth and invasion by suppressing BDNF. Oncotarget. 2017; 8:2825–34. 10.18632/oncotarget.13747 27926508 PMC5356845

[20] Zhuang J, Ye Y, Wang G, Ni J, He S, Hu C, Xia W, Lv Z. MicroRNA-497 inhibits cellular proliferation, migration and invasion of papillary thyroid cancer by directly targeting AKT3. Mol Med Rep. 2017; 16:5815–22. 10.3892/mmr.2017.7345 28849051 PMC5865779

[21] Wang L, Jiang CF, Li DM, Ge X, Shi ZM, Li CY, Liu X, Yin Y, Zhen L, Liu LZ, Jiang BH. MicroRNA-497 inhibits tumor growth and increases chemosensitivity to 5-fluorouracil treatment by targeting KSR1. Oncotarget. 2016; 7:2660–71. 10.18632/oncotarget.6545 26673620 PMC4823062

[22] Raj N, Bam R. Reciprocal crosstalk between YAP1/hippo pathway and the p53 family proteins: mechanisms and outcomes in cancer. Front Cell Dev Biol. 2019; 7:159. 10.3389/fcell.2019.00159 31448276 PMC6695833

[23] Shibata M, Ham K, Hoque MO. A time for YAP1: tumorigenesis, immunosuppression and targeted therapy. Int J Cancer. 2018; 143:2133–44. 10.1002/ijc.31561 29696628 PMC6540999

[24] Celano M, Mignogna C, Rosignolo F, Sponziello M, Iannone M, Lepore SM, Lombardo GE, Maggisano V, Verrienti A, Bulotta S, Durante C, Di Loreto C, Damante G, Russo D. Expression of YAP1 in aggressive thyroid cancer. Endocrine. 2018; 59:209–12. 10.1007/s12020-017-1240-6 28120182

[25] Liao T, Wen D, Ma B, Hu JQ, Qu N, Shi RL, Liu L, Guan Q, Li DS, Ji QH. Yes-associated protein 1 promotes papillary thyroid cancer cell proliferation by activating the ERK/MAPK signaling pathway. Oncotarget. 2017; 8:11719–28. 10.18632/oncotarget.14319 28036290 PMC5355298

[26] Wu DM, Wang S, Wen X, Han XR, Wang YJ, Shen M, Fan SH, Zhang ZF, Shan Q, Li MQ, Hu B, Lu J, Chen GQ, Zheng YL. LncRNA SNHG15 acts as a ceRNA to regulate YAP1-hippo signaling pathway by sponging miR-200a-3p in papillary thyroid carcinoma. Cell Death Dis. 2018; 9:947. 10.1038/s41419-018-0975-1 30237435 PMC6148237

[27] Li R, Liu J, Li Q, Chen G, Yu X. miR-29a suppresses growth and metastasis in papillary thyroid carcinoma by targeting AKT3. Tumour Biol. 2016; 37:3987–96. 10.1007/s13277-015-4165-9 26482618

[28] Chen X, Xiong D, Ye L, Wang K, Huang L, Mei S, Wu J, Chen S, Lai X, Zheng L, Wang M. Up-regulated lncRNA XIST contributes to progression of cervical cancer via regulating miR-140-5p and ORC1. Cancer Cell Int. 2019; 19:45. 10.1186/s12935-019-0744-y 30858762 PMC6394057

